# Bacteriophage Challenges in Industrial Processes: A Historical Unveiling and Future Outlook

**DOI:** 10.3390/pathogens13020152

**Published:** 2024-02-07

**Authors:** Bartosz Kamiński, Jan Paczesny

**Affiliations:** Institute of Physical Chemistry, Polish Academy of Sciences, Kasprzaka 44/52, 01-224 Warsaw, Poland; bkaminski@ichf.edu.pl

**Keywords:** contaminations, bacteriophages, infections, fermentation, industry, bacteria, antiphagents

## Abstract

Humans have used fermentation processes since the Neolithic period, mainly to produce beverages. The turning point occurred in the 1850s, when Louis Pasteur discovered that fermentation resulted from the metabolism of living microorganisms. This discovery led to the fast development of fermented food production. The importance of industrial processes based on fermentation significantly increased. Many branches of industry rely on the metabolisms of bacteria, for example, the dairy industry (cheese, milk, yogurts), pharmaceutical processes (insulin, vaccines, antibiotics), or the production of chemicals (acetone, butanol, acetic acid). These are the mass production processes involving a large financial outlay. That is why it is essential to minimize threats to production. One major threat affecting bacteria-based processes is bacteriophage infections, causing substantial economic losses. The first reported phage infections appeared in the 1930s, and companies still struggle to fight against phages. This review shows the cases of phage infections in industry and the most common methods used to prevent phage infections.

## 1. Introduction

Viruses are microorganisms on the edge of living and non-living matter. They multiply and undergo evolution but do not have a metabolism and are very simple in structure. Researchers estimate that there are 10^31^ virions on Earth at any given moment, with bacteriophages being the most common [1]. Bacteriophages, also called phages, are viruses that infect bacteria. They are composed of two major types of molecules, which sometimes are sourounded by a lipid envolope. The first are proteins, which form different structural parts of a viral particle, like the head, tail, fibers, and sometimes spikes. These proteins allow for phages to detect the host bacterial cells and interact with them. While the variety of shapes facilitates the connection with the host cell and its infection, the head of a phage is where the genome is stored. The genome is a nucleic acid (RNA or DNA). Phages need the host’s cell to replicate. The infection causes multiplication and eventual release of progeny virions, usually resulting in the death of the host cell. This makes phages natural antibacterial agents.

## 2. Taxonomy of Phages

**Figure 1:**
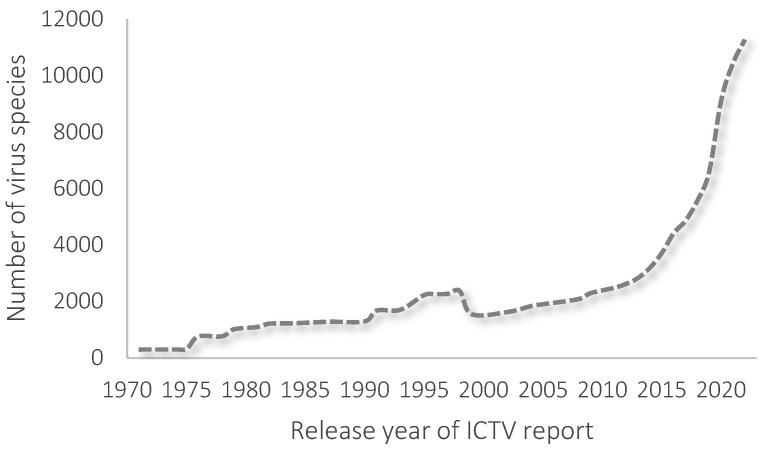
Changes in the number of species of viruses in each report of ICTV.

Viruses are peculiar because of the consistent features of living and non-living matter. That is what makes the taxonomy of viruses hard to develop. The International Committee on Taxonomy of Viruses (ICTV) is responsible for classifying species of viruses, including phages. ICTV publishes updated reports around once a year. Changes in the number of known viruses published in ICTV reports are presented in Figure 1. The last report included 6 realms, 10 kingdoms, 17 phyla, 40 classes, 72 orders, 264 families, 182 subfamilies, 2818 genera, and 11,273 species of viruses.

In the last report of ICTV, 174 proposals were approved [2]. The changes mainly involved renaming virus species using the binominal format. In this convention, the full name of a virus consists of the genus name and species epithet (e.g., *Escherichia virus T4*, renamed to *Tequatrovirus T4*). This way of naming each species was voted on in 2020 [3]. A total of 6481 of the 10,434 virus species have been renamed in a binominal format. Another change was to ratify seven new orders and 48 new families.

We differentiate several types of phages based on their genome and morphology. Most phages’ genomes consist of linear dsDNA, such as *Herelleviridae*, *Myoviridae*, or *Straboviridae*. Some phages have genomes in the form of circular dsDNA (e.g., *Corticoviridae*, *Intestiviridae*, or *Matshushitaviridae*) and circular ssDNA (e.g., *Finnlakeviridae*, *Inoviridae*, *Microviridae,* and *Plectoviridae*). There are also phages with linear ssRNA (*Fiersviridae* and *Duinviridae*) and linear dsRNA (*Cystoviridae*). The prefix ds stands for double-stranded, and ss stands for single-stranded.

**Figure 2:**
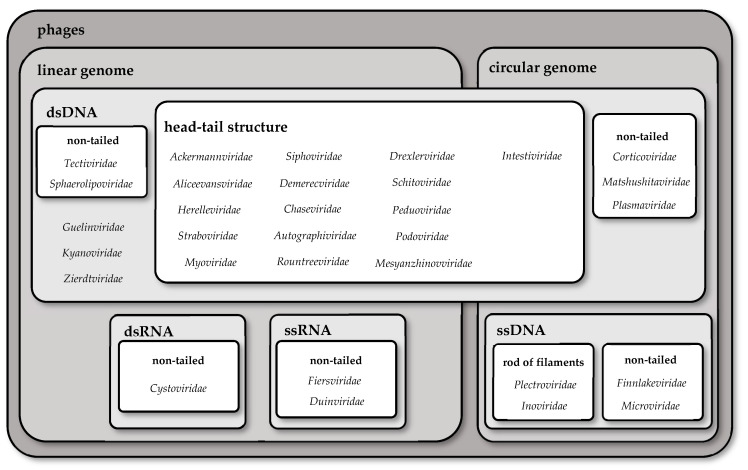
Differences in the morphology of chosen families of phages.

Phages have various morphologies. Some of them have an additional lipid layer on the surface; we call these enveloped phages, e.g., *Plasmaviridae* and *Cystoviridae* [4]. However, most of the phages are non-enveloped. We can also highlight non-tailed phages (e.g., *Sphaerolipoviridae* or *Tectiviridae*) and phages with a head–tail structure. The second group is divided into phages with long, contractile tails (e.g., *Ackermannviridae*, *Mesyanzhinovviridae,* and *Peduoviridae*), with long, non-contractile tails (e.g., *Drexlerviridae*, *Siphoviridae* or *Chaseviridae*), and with short, non-contractile tails (e.g., *Autographiviridae*, *Podoviridae* or *Schitoviridae*). There are also phages with the shape of rods or filaments (*Inoviridae* and *Plectroviridae*). In 2006, Ackermann reported that at least 5,568 phages had been observed under the electron microscope since 1959 [5]. According to his findings, more than 96% of phages possess tails [5]. The differences in the morphology and genome structure of phages are summarized in Table 1 and Figure 2.

## 3. Mechanisms of Infections

The infection of bacteria cells by phages follows lysogenic and/or lytic mechanisms. In the lytic cycle, phages adsorb on the surface of bacterial cells using receptor-binding proteins (RPBs) [6]. Then, the phage introduces its genome into the host cell. Viral material is expressed and replicated using the molecular machinery of bacteria, forming from dozens to thousands of replicated phage particles (virions) inside each host cell. The process ends with the host cell’s lysis and phages’ release into the environment. This life cycle is characteristic of phages like T4 or T7.

The main feature of the lysogenic cycle is the introduction of viral genetic information into the bacterial genome. This allows bacteria to live and reproduce, including the virus’s genes, which we call “prophage”. The phage is in a dormant state. The prophage can be transmitted to other bacteria cells, spreading the viral genome. The whole cycle is harmless for a host cell. However, the prophage is reactivated in the proper conditions, starting the lytic life cycle [7]. The process is called phage switching. Bacteria cells are fragile to temperature, pH, UV light, heavy metals, antibiotics, salt concentration, and chemical oxygen demand. These factors can cause physical or chemical stress that harms the bacteria genome. This activates switching, starting the lytic process [8]. There is also a so-called genetic switch [9]. Phages can insert genes coding information about lysis into bacteria genomes. The expression of genes causes the activation of the lysis mechanism. Chang et al. described the process involving two antagonistic proteins: one acts as a lysis repressor, while the second causes the inactivation of the first protein, leading to lysis [10].

There are some relatively rare examples of chronic infections where viral particles are produced continuously (e.g., the Ff filamentous phages of *E. coli*) [11]. In this mechanism, phages can be produced without killing the bacteria cell. Filamentous phages are secreted from the host cells without disrupting them.

The other rare replication mode is pseudo-lysogeny [12]. This infection can occur because of unfavorable growth conditions, leading to a lack of energy in bacteria cells. Under this type of stress, pseudolysogenic phages (e.g., T4, F116, or UT1) can attack the bacteria. However, due to bacteria’s lowered metabolism, phage DNA’s multiplication and replication also ceases. In more favorable conditions for bacteria, the phage is also activated, leading to the multiplication and replication of the viral genome.

## 4. Fermentation Industry

**Figure 3:**
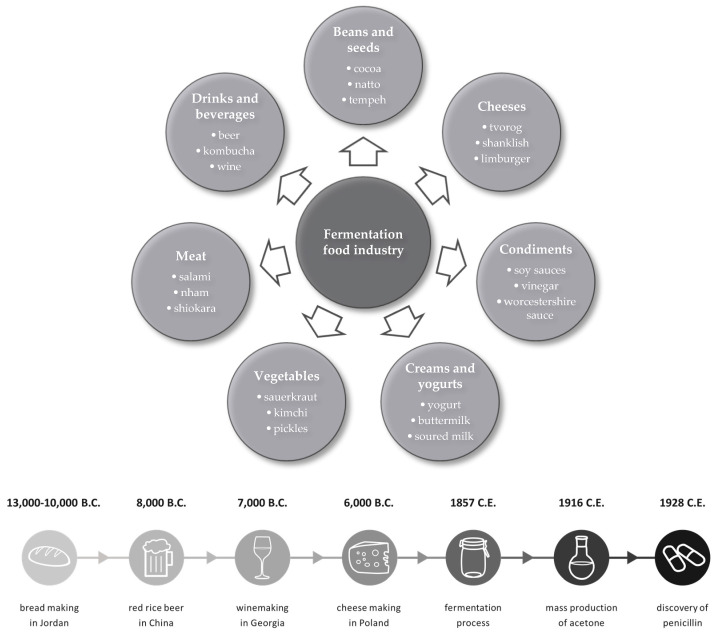
Branches of food industries using fermentation processes and examples of particular products from each [15], along with the timeline of usage fermentation process since pre-Neolithic [13,16,17,18,19,20].

Humans have used microorganisms for thousands of years. The oldest product of fermentation in history is bread. The procedure, i.e., mixing water with flour, leaving it to allow for fermentation, and baking it, is quite simple. There is a report dating bread making to 14.6–11.6 ka cal BP (calibrated years before the present), around 4000 years before the Neolithic [13]. The oldest records of brewery come from 9000 cal. BP, in the Shangshan Culture located along the lower Yangtze River in China. The evidence shows red rice beer production using rice and mold [14]. Fermentation processes involving bacteria and yeast led to the production of other types of bread, beer, wine, and dairy products such as cheese or yogurts. Fermentation has also been an excellent way to extend the shelf life of food, such as pickled vegetables. The most common foods produced through fermentation processes are presented in Figure 3.

The discovery of the mechanism of the fermentation process was reported in 1857 by Louis Pasteur [21]. This was a turning point in industry, affecting the development of industrial fermentation processes. Soon, production based on acetone–butanol fermentation became significant, especially in the context of producing ammunition during World War I [22]. Then, the first antibiotics, penicillin and streptomycin, were discovered, and the fermentation process using *Penicillium chrysogenum* and *Streptomyces griseus* facilitated the large-scale production of these medications [23]. The discovery of penicillin caused the development of the medical industry and led scientists to desire to discover new antibiotics and drugs. At present, fermentation processes are also used in the industrial production of amino acids [24], enzymes [25], and other chemicals [26]. Many drugs (e.g., insulin [27]) are produced using genetically modified organisms, often bacteria.

## 5. Phage Infections

Phage infections are a significant threat to large-scale industrial processes involving bacteria-driven fermentation. Even a small number of phages in fermentation tanks can cause a severe decrease in or failure of the process. Moineau and Lévesque said that large-scale milk fermentations, which start after inoculating around 10^7^ cells per mL of selected lactic acid bacteria, are compromised when the critical threshold for phages is reached, i.e., around 10^4^ plaque-forming units (PFU) per mL, with failure of the process occurring beyond 10^5^ to 10^6^ PFU per mL [28]. It also depends upon the species of phages, the multiplicity of infection, the chosen starters, the composition of the culture medium, and general conditions in fermentation tanks [22]. That is why learning from mistakes and implementing more solutions to protect production processes and prevent such infections is essential. Unfortunately, companies are not likely to share the details of their failures during production. This is certainly understandable: companies do not want to seem like they produce bad-quality products or do not want to admit to substandard conditions. Here, we present reported cases of phage infections throughout history.

### 5.1. 1920s

One of the most critical industrial processes using fermentation was the production of acetone, which Chaim Weizmann developed at the beginning of the 20th century [22]. The process involved the bacterium *Clostridium acetobutylicum*, known as a “Weizmann organism”. His studies became significant during World War I because of the importance of producing explosive materials used during the war. Weizmann was involved in the work with the British government, which allowed him to use his process on a mass scale. His work was expanded to other countries, like Canada and the USA. After the war, butanol gained importance, finding application in different industries. In 1919, the Commercial Solvents Corporation (CSC) set up a plant in Terre Haute, Maryland, where butanol production was the primary goal. The company obtained the worldwide patent rights to the Weizmann process. However, in 1923, “*bacteriological troubles*” were reported. This resulted in fermentation yields being cut in half for about a year. Many researchers have unsuccessfully tried to investigate the problem [29]. To avoid financial losses in production, the company decided to build more fermenters (from 40 to 52) and a new plant 200 miles away, in Peoria, Illinois. A few years later, the case was identified as a phage infection [30]. The first isolation of phage in a butyl fermentation plant was reported by McCoy et al. in 1944 [31]. These investigations led to patenting procedures to produce industrial strains immunized against phage infection [19].

### 5.2. 1930s

At the beginning of the 20th century, cheesemaking relied on undefined bacteria starter cultures. In the early 1930s, Whitehead and Cox isolated one specific strain of bacteria with suitable properties for cheese production: *Lactic streptococci* (known as *Lactococcus lactis*). That was a very successful discovery. However, it turned out that production based on a single strain was problematic. At some point, the starters stopped producing lactic acid, causing the whole production process to fail. In 1935, Whitehead and Cox also reported discovering the first phages specific for the *L. lactis* strains [32]. This was the first description of phages affecting a dairy starter culture. The presence of phage in a starter culture was first recognized during a renewed investigation of the failure in activity caused by the aeration of the milk.

More and more problems with the production yield were reported. One of the cases was the lysis of an actinomycetes culture, described by Dmitrieff and Souteeff [33,34]. A culture of *Actinomyces bovis* was investigated. It was reported that some bacteria overcame the lysis process. That is why they were not able to form an aerial mycelium. Bacteria that had not been lysed could form this mycelium. Researchers did not discover what caused the lysis of the cultures. The only conclusion was that this process had to be associated with “*the living organism and was of the nature of a nonenzymatic byt nontransmissible lytic factor*”. Today, this seems likely to be another example of bacteriophage infection. In parallel, Wieringa and Wiebols (1936) [35,36] also reported the lysis of actinomycetes isolated from infected potatoes. The theory was that this could result from producing “specific transmissible phages”. More detailed research was conducted in 1938 by Krassilnikov [37], who investigated the autolysis of actinomycetes isolated from the soil. The study showed that autolysis appeared not on the whole surface of the colony, but on several spots. This was a time during which a theory about transmissible lytic agents, like bacteriophages, was formed.

### 5.3. 1940s

Phage infections were not only a problem for *S. griseus*. The first phages of *Clostridium madisonii* were isolated in 1943 [31,38]. Abnormal acetone–butanol fermentations was reported in several plants in Japan due to phage contamination, including other species, such as *Clostridium acetobutylicum* [39] and *Clostridium saccharoperbutylacetonicum* [40,41]. Problems with phage infection were also documented in an industrial fermentation process in Puerto Rico in 1943 [31].

In 1947, Saudek and Coligsworth reported the contamination of streptomycin production in Upjohn plants [42,43]. Their research showed the formation of plaques on solid media and the absence of streptomycin in liquid media in the broth of the *S. griseus* strain. These were taken as proof of the lytic activity of phages due to contamination of the production. Soon, the actinophage was isolated from the streptomycin fermentation and then examined [44]. Further research confirmed that phage is active against *S. griseus*, causing decreased streptomycin production. While most phages are specific against a limited number of strains [45], actinophage was first described for phages that are active against other strains of *S. griseus* [46]. Reilly et al. demonstrated that the examined phage was attacking only streptomycin-producing strains of *S. griseus,* and not influencing other streptomycin-producing strains, such as *S. bikiniensis*. Streptomycin production by *S. griseus* was the first example of a large-scale bacterial fermentation to produce a human pharmaceutical [47,48]. In 1950, the Squibb company (New York, NY, USA) also reported several phage infections of *S. griseus* broth in their plants and laboratories [49]. The effect of the infections was low streptomycin potencies.

### 5.4. 1950s

In 1951, a failure in the production of Aureomycin by the American Cyanamid Company (Bridgewater, NJ, USA) was reported. This antibiotic consists of chlortetracycline, a drug for treating infected allergic dermatitis in humans [50]. The roots of the problems were confirmed in 1961 by isolating a phage specific for *S. aureofaciens* [51].

Another massive case of contamination was observed in 1952 [52]. The fermentation process in tanks containing *S. griseus* was marked as “*increased fluidity, diminution or absence of mycelial growth, little or no streptomycin, the presence of a dark brown soluble pigment*”. Further research confirmed phage infection. However, it turned out that the contamination did not come from the stock culture collection, so phages had to be introduced during the process. Almost 67% of the total, 74 out of the 111 examined tanks, were infected. The amount of phage particles ranged from one hundred to trillion particles per mL.

In 1959, Hongo et al. isolated several new strains of the solvent-producing genus *Clostridium*, which produced high levels of butanol [38,53]. However, during the first year in which these new strains were introduced, phage infections continued to cause problems during the industrial fermentation operation [54].

### 5.5. 1960s

In the 1960s, Hongo and coworkers reported serial phage infections in acetone–butanol–ethanol plants throughout Japan [54]. Phages were attacking strains of *Clostridium saccharoperbutylacetonicum*. The infections caused a critical decrease in production efficiency. There were around 12 phage outbreaks in one year. Researchers claimed that the isolation of phage-resistant mutants after each outbreak was unsuccessful; another phage attacked each isolated bacteria. There are no data on the losses of the Japanese plants during this time, but they seemed insurmountable.

*Streptomyces mediterranei* (known as *Amycolatopsis rifamycinica*) is another example of a bacteria used in industrial processes. It is exploited to produce rifamycin antibiotics against mycobacteria to treat tuberculosis, leprosy, and mycobacterium avium complex infections [55]. In 1962, phage contamination was reported by Thiemann et al. [56]. The strains of *S. mediterranei* were attacked by several types of actinophage. Here, we have a case similar to that with the Japanese plants. After each phage outbreak and the isolation of phage-resistant mutants, another phage attacked the strains. Phages isolated from the contaminated broth were identified as β, γ, 17, 112, and 156, as mentioned by the author. The study showed that the size and shape of the observed plaques differed for each phage. However, the authors also did not report any information about the loss in production.

### 5.6. 1970s

In 1971, Whitman and Marshall reported vast contamination in local markets [57]. Samples of refrigerated products, such as beef, chicken, pork, milk, and oysters, were examined. Researchers isolated 38 phages from almost 50% (22 of 45) of the analyzed samples. The researchers stated that refrigerated food products are not free of phages. At present, it is known that phages might increase the shelf life of products by limiting the growth of bacteria. The specific usage of phages in the food industry was recently reviewed by Vikram et al. [58].

In December 1974, the FDA also reported the contamination of vaccines approved for human use in the United States, including polio, mumps, measles, and rubella vaccines [59]. The research showed that 18% (11 out of 60 lots) tested positive for coliphages, from 1 to 5 PFU/ml. the observed plaques were similar to those observed in bovine serum. In other research, scientists confirmed the isolation of three species of phages: ϕV-1, ϕV-2, and ϕV-3 [60].

These events forced the FDA to stop releasing new vaccines for two weeks [61] and they stated that pharmaceutical products should not include any additional external agents, including phages. These decisions deeply changed the pharmaceutical industry. Many companies were scared about possible new guidelines and regulations from the FDA [62,63]. The production of some vaccines uses fetal bovine serum, which goes through filtration to obtain samples that are clean of bacteria. However, the used filters allow for phages to pass through. Using sterile bovine serum can cost two times more, causing costs to increase from 60 USD to 120 USD per liter.

There was a vivid debate about the implications of phages’ presence in vaccines. Some scientists believe that infected bacteria can produce toxins that harm humans. The most known example is diphtheria, a disease caused by the bacterium *Corynebacterium diphtheriae* after phage infections. Phages introduce genetic information about the toxin’s structure and the genes that are expressed, harming bacteria and humans [64]. Another possible threat is the transmission of phage genes into human cells and synthesizing of viral proteins, which may cause some diseases. Merrill et al. reported the ability to introduce genes expressing galactose-1-phosphate uridyltransferase via lambda phages in cells [65]. It was also shown that phages like T7 can penetrate eukaryotic cells in Syrian hamsters, and be transported to the nucleus [66]. A recent review on toxins related to phages was published by Casas and Maloy [67]. However, scientists assume that people are exposed to phages daily, and the exact implications of this are unknown. Recent studies by Dąbrowska and coworkers showed antibodies against T4-like phages were present in 80% of the population [68]. Moreover, phages are also used as therapeutic agents, for example, in curing liver diseases [69], diabetic foot infections [70], or even cancer [71].

Ultimately, the FDA allowed companies to produce vaccines containing phages [61]. Phage ϕV-1 was chosen for further studies. There was no evidence of any influence on the genome of examined cells [72]. This case was critical in facing the problem of the influence of phages on eukaryotic cells and developments in the field of phages’ interactions with humans.

In 1972, the National Institute of Mental Health reported that commercially produced sera in four companies could be contaminated with mycoplasmas and bovine viruses. Further research showed that characteristic plaques were observed in some examined sera samples. The research reported that this was caused by bacteriophages in those samples, even 2270 PFU/ml [62]. In July 1973, this study was also confirmed by the Food and Drug Administration (FDA) [73]. In 85% of the examined samples (23 out of 37), bacteriophages of *E. Coli* were detected. In this research, the PFU per mL was between 1 and 10^4^.

In 1977, Sozzi et al. examined samples of by-products obtained from cheese and butter plants from Columbia and Spain. The researchers stated that all bacterial strains are vulnerable to phage infection in industrial conditions because of the large quantities of fermentation broth (thousands of liters a day) [74]. A similar conclusion was stated by Lawrence in 1978: the phage causes the lysis of bacteria to occur more quickly when introducing new strains at an industrial scale [75].

### 5.7. 1980s

In 1982, milk fermentation contamination of *Lactobacillus casei* strain S-1 was reported at the Yakult Central Institute for Microbiological Research in Japan. The phage responsible for the abnormal fermentations was φFSV. This phage originated from prophage φFSW and was its virulent mutant. φFSV became virulent after changing the location of an insertion element [76].

Phage infections also threatened a winery, where malolactic fermentation is a crucial step. In 1985, Davis et al. isolated *Leuconostoc oenos* phages (known as *Oenococcus oeani*) from red wine production in one Australian winery [77]. A total of 25% of samples (4 of 16) from the investigated processes tested positive for phages. However, there were very few studies about the dangers of phage infections of *Leuconostoc* bacteria, in contrast to lactic acid bacteria in the dairy industry [78,79,80]. This was important because *Leuconostoc* bacteria became increasingly popular in wine production in the 1980s.

In 1983, unexplained problems were also reported in Germany during submerged vinegar fermentations using acetic acid bacteria: a sudden decrease in product yield and changes in the optical density of bacteria samples were observed [81]. Further investigation confirmed contamination with phages at level 10^9^ viral particles per mL [82]. The analysis made it clear that phages were to blame for the production failure, as they induced the lysis of bacteria cells. This research first describes vinegar fermentations as being vulnerable to phage infections. That was the beginning of troubles with phages in the vinegar industry, which continued in the 1990s.

Until the end of the 1980s, there were only four known phages of acetic acid bacteria, i.e., *Acetobacter* phage AA1 (known as *Aeromonas* phage) [83,84], phage A-1 [84], phage GW6210 [85], phage JW2040 [85], and phage MO1 [86]. Infections in this branch of industry were rare. However, in 1989, Stamm et al. reported unexplained failures in vinegar fermentations in several European countries, i.e., France, Switzerland, and Germany [87]. Because of those events, the French company incurred substantial economic losses because of repetitive spontaneous phage outbreaks. Many European vinegar factories appeared to struggle with bacteriophage infections, reaching 10^8^–10^9^ particles per mL of fermentative broths. Even disinfection with hydrogen peroxide was not very helpful. Stamm et al. isolated and identified two types of phage: type I, a phage with contractible tails, similar to phages A-1 and MO1; and type II, similar to JW2040 phage.

### 5.8. 1990s

In 1992, Sellmer et al. investigated 300 samples from companies that reported breakdowns during vinegar fermentations with *Acetobacter europaeus*, placed in Austria, Denmark, and Germany [88]. The research showed that 70% of samples (210 of 300) contained phages, reaching from 10^7^ to 10^9^ particles per mL of broth. The researchers described seven new types of phages with different head sizes, tail lengths, and general morphologies. The authors suggested using pasteurized substrates, sterile conditions, and separating trickle and submerged fermentation tanks [88].

In 1992, another phage infection was reported [89]. During industrial fermentation, a breakdown was observed in the Czech and Slovak Federal Republic. The process of amino acid production utilizing *Brevibacterium flavum* was contaminated with phage BFK20.

Moineau et al. isolated 27 lactococcal-specific phages. The samples were delivered from 27 cultured buttermilk plants from different parts of the USA [90]. A total of 80% of isolated phages (22 of 27) were 936-type phages; the rest were P335 and c2 species. Researchers also pointed to the increasing frequency of P335-type phages in factories worldwide, including Canada [91], Denmark [92], and New Zealand [93]. Scientists stated that an emergency regarding this kind of phage might have appeared in industry only a few years before the study.

Until 1997, the Swiss company Nestle collected 80 examined phages tat were harmful to the dairy industry [94]. They were all delivered by European plants and factories where fermentation breakdowns occurred, mainly producing yogurts and cheese. The company’s report stated that a more extensive diversity of phages was isolated from cheese factories than yogurt and pointed out that raw milk is a critical source of phages.

Gindreau and Lonvaud [95] reported another case of phage infection in the wine industry. Abnormal malolactic fermentation caused a decrease in the acidity and general quality of wine. The researchers pointed to *Oenococcus oeni* phages, such as φ10MC, as a primary threat regarding this kind of failure in winemaking.

The production of 2-keto-D-gluconic acid (2KGA) is executed at an industrial scale and involves several bacteria, i.e., *Gluconobacter*, *Pseudogluconobacter*, or *Pseudomonas* [96]. 2KGA is a crucial precursor used to synthesize vitamin C [97]. Chemical synthesis consumes vast quantities of toxic platinum and lead catalysts, has low selectivity, and generates enormous costs. That is why a biotechnological path using fermentation seems to be a better option. However, the bacteria-driven process is vulnerable to phages. Severe infection was reported in 1999 in several Chinese plants. During these events, five new phages were isolated and characterized.

### 5.9. 2000s

From 1994 to 2000, extensive research on phages was carried out in Argentina. *Programa de Lactologia Industrial PROLAIN* was a program to investigate the phages that can be found in Argentinian plants and factories [98]. There were 129 samples from yogurt and cheese plants, with 96 and 33, respectively, for each. In the results, 61 phages were isolated: 59 were phages of *S. thermophilus*, and 2 were phages of *L. delbrueckii*. A total of 18% of yogurt samples and 67% of cheese samples were contaminated. The number of phage particles varied from 10^2^ to 10^9^ PFU per mL, depending on the product and location.

In 2004, Madera et al. investigated a significant number of raw milk samples from plants located in an area of 10,500 km^2^ in northern Spain [99]. The researchers examined 900 samples of raw milk. A total of 9.2% (83 of 900) samples were contaminated with phages, while the most common was c2-like phages. This large-scale investigation confirmed that raw milk is the primary source of contamination with phages for *L. lactis* in dairy factories.

Moineau and Lévesque compiled various historical instances of phage infections affecting fermentation [28]. Their summary, published in 2004, was updated compared to that of Ackermann and DuBow (1987) [100], Jones et al. (2000) [22], Ogata (1980) [101], and Wünsche (1989) [102]. The authors listed the infected bacterial species used for the production of food (e.g., vinegar, fermented soybeans, fermented milk, yogurt, cheese, sourdough bread, silage, sauerkraut, buttermilk, sour cream, soy sauce), drugs (e.g., colistin, polymycin, kanamycin, chloramphenicol, streptomycin, endomycin, tetracycline), insecticide, chemicals (acetone, butanol) and other biotechnology products (e.g., L-glutamic acid, gluconic acid, lactic acid, 2-keto gluconic acid, xantham). These examples were not described in more detail (date, location, magnitude of the problem), as the authors focused on protection against phages and the detection of phages.

In 2006, Zhang et al. investigated 20 yogurts produced in small factories in different Chinese cities [103]. The presence of phages was confirmed using the spot and turbidity tests. From the 20 yogurt samples, researchers isolated 20 *Lactobacillus* strains. A total of 35% of examined strains (7 of 20) turned out to be lysogenic. Scientists stated that contamination in small Chinese factories uses low-quality substrates.

There was also communication about phage contamination in 2006 during the *E. coli*-mediated production in NutraSweet^®^ (Chicago, IL, USA), an American company that produces neotame, an artificial sweetener [104,105].

### 5.10. 2010s

In 2010, a new phage infection was reported. Pringsulaka et al. examined samples from a Thai factory producing Nham, a traditional, uncooked, fermented sausage made of pork, rice, and some seasoning [106]. *Lactobacilli* and *Pediococci* bacteria drive the fermentation. The case is the first-ever report about phage infection in the meat industry in Thailand. The authors investigated 39 samples of Nham from northern Thailand. The study showed a new *Podoviridae* lactic acid bacteria phage: phi22. The researchers focused on characterizing the phage without investigating the causes of the infection. They stated that phi22 is active against the starter culture used in Nham fermentation. Other researchers suggest using a mixed starter culture in the future.

## 6. Future Outlook: New Antiphagents

The cases described in previous chapters prove that phage infection in industrial processes is a huge problem, even now. One of the primary strategies, especially in the dairy industry, is using multi-species starter cultures, which contain several types of bacteria, each with a different phage sensitivity and/or resistance [107,108,109,110]. This allows for the prevention of complete failure of production. Because of their high specificity, phages can attack one species of bacteria, so using more phage can minimize losses in the output. Rotation of the starter culture is also a good strategy [111]. The company can achieve a relatively high production yield if only one bacteria species is used a starter culture. This might also be achieved by using concentrated cultures for inoculation.

It is also essential to design the process and choose the proper equipment to drive the industrial process and keep it clean and airtight. Various procedures and protocols are crucial for maintaining safe conditions. However, the presence of phages is sometimes inherent to the process, especially when substrates used for the production are of natural origin. The sheer number of virions in the environment results in a relatively high risk of contamination, with phages able to infect the bacteria used in the process. Sometimes, there is no good way to sterilize additives, e.g., antibiotics. Some treatments (e.g., heat) result in a loss of activity not only in the contaminants (i.e., phages) but also in vital components of the process. Thus, phage detection is crucial. When the phages are present, novel antiphagents (anti-phage agents) are needed. Although there are many ways to prevent phage infections, they are still a massive threat to all bacteria-driven industrial-scale processes. Developing new methods for protection against phages will be crucial in the following years. The most commonly used physical and chemical methods are listed and described in a review by Raza et al. [112].

### 6.1. Physical Factors

Phages are mainly made of proteins. It is reported that increasing the temperature to 65–72 °C leads to the disintegration of the protein virion-containing genome of the phage [113,114,115]. A temperature of 72 °C is required to destroy the MS2 virions due to the release of the genome [116]. However, not all phages are sensitive to thermal treatment [117]. Higher temperatures are also not very likely to be used in the dairy industry because of the possible product damage [118].

Pressure is another physical factor that can cause the inactivation of phages, especially in industry branches that cannot use higher temperatures. Several techniques have been developed using pressure, such as high hydrostatic pressure (HHP), high-pressure homogenization (HPH), and dynamic high pressure (DHP). Some researchers showed that a pressure of 300 MPa can decrease the number of phages by 99% [119]. However, not all phages are still vulnerable to high pressure, with *E. coli* phages Qβ and c2 being examples that are able to survive 500–700 MPa for 30 minutes [120].

Radiation also can help to work against phages. UV radiation causes the formation of free radicals that can destroy the phage genome [121,122]. Even near-UV radiation was reported as being effective [123]. This method is not applicable in many instances because of the low penetration depth and geometrical obstacles. Other methods utilize an electric field, especially in the form of pulses (pulse electric field (PEF)) [124] and osmotic shock [125,126].

### 6.2. Chemical Factors

In addition to the physical factors, there is a fast-developing branch of synthesizing antiphagents, i.e., agents that work against the phages without harming bacteria. A variety of compounds can act as antiphagents. Several reports show the antiviral properties of polymers, such as ε-poly-L-lysine, which was found to be effective against T4 and T5 phages [127]. α-poly-L-lysine [128,129] and poly-D,L-alanine [127] were also studied in this context. Very recently, Marton et al. showed that poly(acrylic acid) prevents phage replication and the phage-induced death of host cells while being neutral to recombinant protein expression [130]. Also, some polysaccharides, such as chitosan [131] and poly(N-2-hydroxyethyl acrylamide) [132], were reported to have antiviral properties.

Other chemicals are also used, i.e., benzalkonium chloride, hydrogen peroxide, alkaline detergent mixtures, or potassium peroxymonosulfate [133,134,135], and commercially available disinfectants like Virkon S, ethanol, and Triclosan [136,137,138]. Potential antiphagents also include natural extracts, e.g., tea [139] and other herbs, like thyme [140], Baikal skullcap [141], or rosemary [142].

It is also reported that heavy-metal salts, i.e., lead [143], mercury [144], copper [145], and cadmium [146] affect virions. Those ions bind to the surface of the phage and then cause structural damage to virions, inactivating the whole phage. However, precious metal salts, like silver [147], gold [148], and platinum [149], also have antiviral properties.

### 6.3. Antiphagents

Nanoparticles can act as antiphagents. The most investigated NPs are based on metals, e.g., silver, copper oxide, and titanium dioxide. Nanoparticles might act via three distinct mechanisms: (i) adsorbing on the surface and destabilizing the protein structure, (ii) releasing ions, and (iii) generating reactive oxygen species [150]. The same mechanisms are also valid while considering the antibacterial properties of nanoparticles. However, nanoparticles, which are safe for bacteria and possess antiviral properties, have finally been reported. Richter et al. [151] presented gold nanoparticles with different ratios of negatively charged (11-mercapto 1-undecanesulfonic acid) and hydrophobic (1-octanethiol) ligands that can cause deactivation through electrostatic attaching to the surface of the virion. Very recently, it was shown that the concentration of ligands at the surface of nanoparticles plays a crucial role in the process [152].

Other research shows exciting properties of iron that make it a possible antiviral agent. The tail contains iron ions, which bind with bacteria and facilitate infection. That is why iron nanoparticles can cause phages to be adsorbed on their surface, causing the deactivation of phages [153]; this is used, e.g., in hematite nanoparticles to remove up to 1.5 logs of MS2 phage [154] or in bio-sand filters containing iron oxide to remove viruses from water [155]. Some Fe-based compounds, like FeSO_4,_ react with proteins in the phage capsid and generate reactive forms of oxygen (ROS), e.g., hydroxyl radicals, through Fenton’s reaction [156]. It was also observed that the higher the concentration of iron ions, the more phages are inactivated [157]. Another interesting example was the zero-valent iron particles [150].

Nanoparticles are a very promising area in this field of research. The assortment of materials that can be used to synthesize nanoparticles’ cores and attach a variety of ligands creates infinite possibilities of obtaining a material with the desired properties that can be easily modulated. This makes nanoparticles excellent candidates for use as antiphagents in industrial processes.

## 7. Summary

In summary, this comprehensive exploration of phage infections in the fermentation industry provides a historical perspective of the significant impact of these viral contaminants. Spanning from the early 20th century to the present, the article elucidates instances of the production setbacks, economic losses, and scientific challenges posed by phage infections across diverse industrial fermentation processes.

The historical narrative traces the evolution of fermentation practices, emphasizing their pivotal role in producing essential commodities such as antibiotics, vaccines, and industrial chemicals. Despite the longstanding use of microorganisms in fermentation, the emergence of phage infections became a recurrent obstacle, affecting processes ranging from acetone production to vaccine manufacturing.

The 1920s witnessed a significant setback in acetone production due to "*bacteriological troubles*", later identified as a phage infection. In the 1930s, phages affecting dairy starter cultures were reported, leading to production challenges. The 1940s saw phage contamination affecting streptomycin production, while the 1950s observed failures in aureomycin production linked to phage infections. In the 1960s, serial phage infections in acetone–butanol–ethanol plants and phage contamination in rifamycin antibiotic production were reported. The 1970s marked a critical period when the FDA reported phage contamination in vaccines, causing a temporary halt in vaccine releases. The ensuing debate centered on the potential implications of phages in vaccines, with concerns about toxin production and gene transfer into human cells. Despite their initial reluctance, the FDA eventually allowed vaccine production containing phages. The 1980s and 1990s witnessed diverse phage infections in milk fermentation, winemaking, amino acid production, and vinegar fermentation. These events caused economic losses, emphasizing the need for preventive measures. Furthermore, more recent cases in the 2000s and 2010s underscore the continued relevance of phage infections in the fermentation industry. Notable examples include incidents in milk fermentation, winemaking, artificial sweetener production, and even the traditionally overlooked meat fermentation sector.

The fermentation industry, rooted in ancient practices, initially utilized microorganisms to produce food and extend shelf life. The pivotal discovery of the fermentation mechanism by Louis Pasteur in 1857 marked a turning point, influencing industrial processes. Now, we depend on bacteria-based processes in numerous branches of industry. Phages pose the biggest threat to these processes; thus, new antiphagents must be developed.

## Figures and Tables

**Table 1:** Differences in chosen families of phages.

Family	Shape of Genome	Nucleic Acid	Structure	Type of Tail
*Ackermannviridae*	linear	dsDNA	head–tail structure	long, contractile tail
*Aliceevansviridae*	linear	dsDNA	head–tail structure	long, contractile tail
*Herelleviridae*	linear	dsDNA	head–tail structure	long, contractile tail
*Straboviridae*	linear	dsDNA	head–tail structure	long, contractile tail
*Myoviridae* ^1^	linear	dsDNA	head–tail structure	long, contractile tail
*Mesyanzhinovviridae*	linear	dsDNA	head–tail structure	long, contractile tail
*Peduoviridae*	linear	dsDNA	head–tail structure	long, contractile tail
*Drexlerviridae*	linear	dsDNA	head–tail structure	long, non-contractile tail
*Siphoviridae* ^1^	linear	dsDNA	head–tail structure	long, non-contractile tail
*Demerecviridae*	linear	dsDNA	head–tail structure	long, non-contractile tail
*Chaseviridae*	linear	dsDNA	head–tail structure	long, non-contractile tail
*Autographiviridae*	linear	dsDNA	head–tail structure	short, non-contractile tail
*Rountreeviridae*	linear	dsDNA	head–tail structure	short, non-contractile tail
*Podoviridae* ^1^	linear	dsDNA	head–tail structure	short, non-contractile tail
*Schitoviridae*	linear	dsDNA	head–tail structure	short, non-contractile tail
*Tectiviridae*	linear	dsDNA	non-tailed	-
*Sphaerolipoviridae*	linear	dsDNA	non-tailed	-
*Guelinviridae*	linear	dsDNA	-	-
*Kyanoviridae*	linear	dsDNA	-	-
*Zierdtviridae*	linear	dsDNA	-	-
*Cystoviridae* ^2^	linear	dsRNA	non-tailed	-
*Fiersviridae*	linear	ssRNA (+)	non-tailed	-
*Duinviridae*	linear	ssRNA (+)	non-tailed	-
*Intestiviridae*	circular	dsDNA	head–tail structure	short, non-contractile tail
*Corticoviridae*	circular	dsDNA	non-tailed	-
*Matshushitaviridae*	circular	dsDNA	non-tailed	-
*Plasmaviridae* ^2^	circular	dsDNA	non-tailed	-
*Microviridae*	circular	ssDNA	non-tailed	-
*Finnlakeviridae*	circular	ssDNA	non-tailed	-
*Inoviridae*	circular	ssDNA	rod of filaments	-
*Plectroviridae*	circular	ssDNA	rod of filaments	-

^1^ Families *Myoviridae*, *Podoviridae*, and *Siphoviridae* were abolished in the recent ICTV report. However, species belonging to these families have not yet been classified into new families, so the informal names *myovirus*, *podovirus*, and *siphovirus* are allowed. ^2^ Enveloped phages.

## Data Availability

Not applicable.
